# Opioid Exacerbation of Gram-positive sepsis, induced by Gut Microbial Modulation, is Rescued by IL-17A Neutralization

**DOI:** 10.1038/srep10918

**Published:** 2015-06-03

**Authors:** Jingjing Meng, Santanu Banerjee, Dan Li, Gregory M. Sindberg, Fuyuan Wang, Jing Ma, Sabita Roy

**Affiliations:** 1 Department of Pharmacology, University of Minnesota Medical School, Minneapolis, Minnesota; 2 Department of Surgery, Division of Infection, Inflammation, and Vascular Biology, University of Minnesota Medical School, Minneapolis, Minnesota; 3 Department of Infectious Diseases, First Affiliated Hospital of China Medical University, Shenyang, China; 4 Department of Veterinary Population Medicine, College of Veterinary Medicine, University of Minnesota, St. Paul, Minnesota

## Abstract

Sepsis is the predominant cause of mortality in ICUs, and opioids are the preferred analgesic in this setting. However, the role of opioids in sepsis progression has not been well characterized. The present study demonstrated that morphine alone altered the gut microbiome and selectively induced the translocation of Gram-positive gut bacteria in mice. Using a murine model of poly-microbial sepsis, we further demonstrated that morphine treatment led to predominantly Gram-positive bacterial dissemination. Activation of TLR2 by disseminated Gram-positive bacteria induced sustained up-regulation of IL-17A and IL-6. We subsequently showed that overexpression of IL-17A compromised intestinal epithelial barrier function, sustained bacterial dissemination and elevated systemic inflammation. IL-17A neutralization protected barrier integrity and improved survival in morphine-treated animals. We further demonstrated that TLR2 expressed on both dendritic cells and T cells play essential roles in IL-17A production. Additionally, intestinal sections from sepsis patients on opioids exhibit similar disruption in gut epithelial integrity, thus establishing the clinical relevance of this study. This is the first study to provide a mechanistic insight into the opioid exacerbation of sepsis and show that neutralization of IL-17A might be an effective therapeutic strategy to manage Gram-positive sepsis in patients on an opioid regimen.

Sepsis and septic shock are the leading causes of death in intensive care units (ICUs) and have significant mortality rates (as high as 60%) and health-care cost burdens (40% of total ICU expenditures)^1^. Recent epidemiological studies have shown that approximately 47% of ICU patients with severe sepsis have positive cultures for Gram-positive bacteria, and the contribution of these bacteria to sepsis has been steadily increasing since 1979^2^,^3^. The clinical manifestation of sepsis is highly variable and influenced by several factors, including the health and immune status of the patient and the infectious agent involved. In the ICUs, where sepsis is a common occurrence following surgery, attention needs to be directed to the hospital-specific attributes, including (but not limited to) altered metabolism and immune-modulation due to opioid administration, to gain mechanistic insights into poly-microbial sepsis and its potential remedy^4^,^5^.

Due to their analgesic and sedative properties, opioids are widely used in ICUs to optimize patient comfort and facilitate mechanical ventilation^6^. The immunosuppressive effects of opioids are well documented^5^ and raise safety issues, especially in ICU patients. In humans, higher circulating morphine levels are observed in patients with sepsis, severe sepsis, and septic shock^7^, and several murine sepsis models show that morphine treatment induces bacterial translocation from the gut lumen into the peritoneal organs and circulatory system^8^,^9^. We have previously shown that morphine accelerates the progression of LPS-induced sepsis by modulating Toll-like receptor (TLR) pathways and altering endotoxin tolerance^10^. However, the exact mechanisms by which opioids modulate sepsis progression remain largely elusive. In the present study, we used cecal ligation and puncture (CLP), a well-established model for inducing poly-microbial sepsis, in C57BL/6J mice treated with either opioids or placebo. The survival rates of mice were analyzed to investigate the effects of opioids on sepsis progression. We demonstrated that both morphine and methadone treatment resulted in high mortality following CLP when compared with placebo-treated animals. Furthermore, morphine promoted bacterial dissemination and increased production of the pro-inflammatory cytokine interleukin-17A (IL-17A).

IL-17A is a member of the interleukin-17 (IL-17) family, which consists of a subset of cytokines that participate in both acute and chronic inflammatory responses. In various diseases, IL-17A is involved in host defense and implicated in excessive inflammation and overt tissue damage^11^. Although a few reports implicate IL-17A in poly-microbial sepsis^12^,^13^, its role in the progression of Gram-positive sepsis is still unknown. Our study showed that overexpression of IL-17A following morphine treatment resulted in increased gut permeability, a higher bacterial load, sustained inflammation, and, subsequently, higher mortality. Concomitantly, neutralization of IL-17A protected morphine-treated animals from sepsis-induced mortality.

We (and others) have previously shown that the source of the bacteria that contributes to morphine-induced sepsis is derived from the commensal pool of the gut microbiome^8^,^9^. In this study, we further showed that morphine treatment induced enrichment of the Gram-positive bacteria *Staphylococcus* and *Enterococcus* in the gut lumen, the same species that were isolated from various systemic organs following CLP. Activation of TLR2 by the disseminated Gram-positive bacteria led to overexpression of IL-17A, resulting in higher mortality in the morphine-treated animals. These results are consistent with the clinical observation that *Staphylococcus aureus* is one of the most common Gram-positive isolates from patients with sepsis, and infection with *Enterococcus* species is considered as an independent factor associated with a greater risk of hospital death^1^.

In summary, the current study provides insight into the influence of opioids on sepsis progression, showing that IL-17A may be a potential therapeutic target for the treatment of sepsis caused by Gram-positive infection, especially in ICU patients who are on a moderate to severe pain management regimen.

## Results

### Opioids increase mortality in a poly-microbial sepsis model of CLP

**Figure 1:**
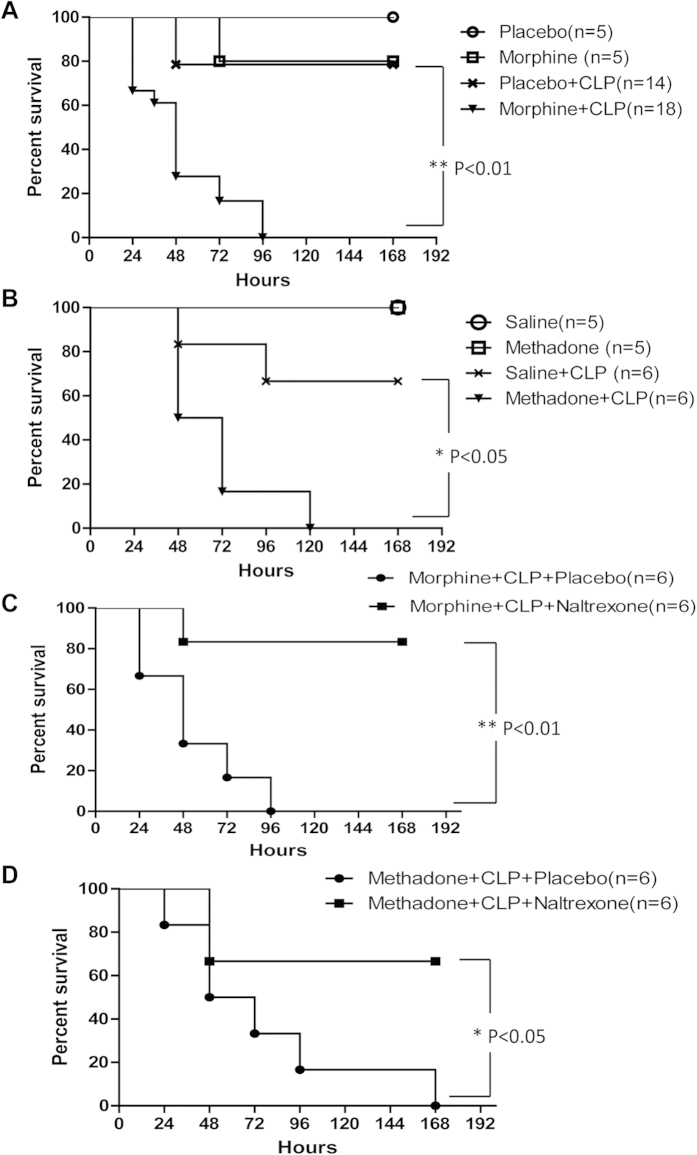
Opioids increase mortality rates in poly-microbial sepsis induced by CLP. (**a**) Kaplan–Meier plots of sham-operated or CLP mice treated with placebo or 25 mg morphine pellet. ** p < 0.01 compared with placebo-treated mice subjected to CLP (Mantel-Cox log rank test) (**b**) Kaplan–Meier plots of sham-operated or CLP mice injected with saline or 15 mg/kg methadone. * p < 0.05 compared with saline-treated mice subjected to CLP (Mantel-Cox log rank test) (**c**) Kaplan–Meier plots of morphine-treated CLP mice treated with placebo or 30mg naltrexone pellet. ** p < 0.01 compared with placebo-treated mice (Mantel-Cox log rank test) (**d**) Kaplan–Meier plots of methadone-treated CLP mice treated with placebo or 30 mg naltrexone pellet. * p < 0.05 compared with placebo-treated mice (Mantel-Cox log rank test) Numbers of mice used for each condition are shown in the frame.

To determine the effects of opioids on the progression of poly-microbial sepsis, wild-type (WT) mice were subjected to a CLP procedure and implanted with either a placebo or a slow release morphine pellet subcutaneously. As shown in Fig. 1a, the survival rates were significantly reduced in morphine-treated mice following CLP. At 24 hours after CLP, all placebo-treated mice were alive compared with only 66.67% in the morphine-treated group. None of the morphine-treated mice survived beyond 96 hours, whereas 78.57% of the placebo-treated mice survived for the entire period of observation (7 days). All of the sham-operated mice treated with the placebo survived, whereas 80% of the sham-operated mice treated with morphine survived until the end of 7 days. To investigate the influence of other prescription opioids on the outcome of CLP-induced sepsis, mice were injected with methadone or saline following CLP. Methadone showed similar effects to morphine on the survival rates following CLP (Fig. 1b). A total of 66.67% of the saline-treated CLP mice lived for 7 days, whereas no methadone-treated mice survived beyond the fifth day after CLP. All of the sham-operated mice injected with saline or methadone survived for 7 days. The morphine- and methadone-induced mortality following CLP was significantly reduced by the opioid receptor antagonist naltrexone (Fig. 1c,d), indicating that opioid treatment exacerbated the outcome of poly-microbial sepsis in an opioid receptor-dependent manner.

### Morphine promotes bacterial dissemination and inhibits bacterial clearance following CLP-induced sepsis

**Figure 2:**
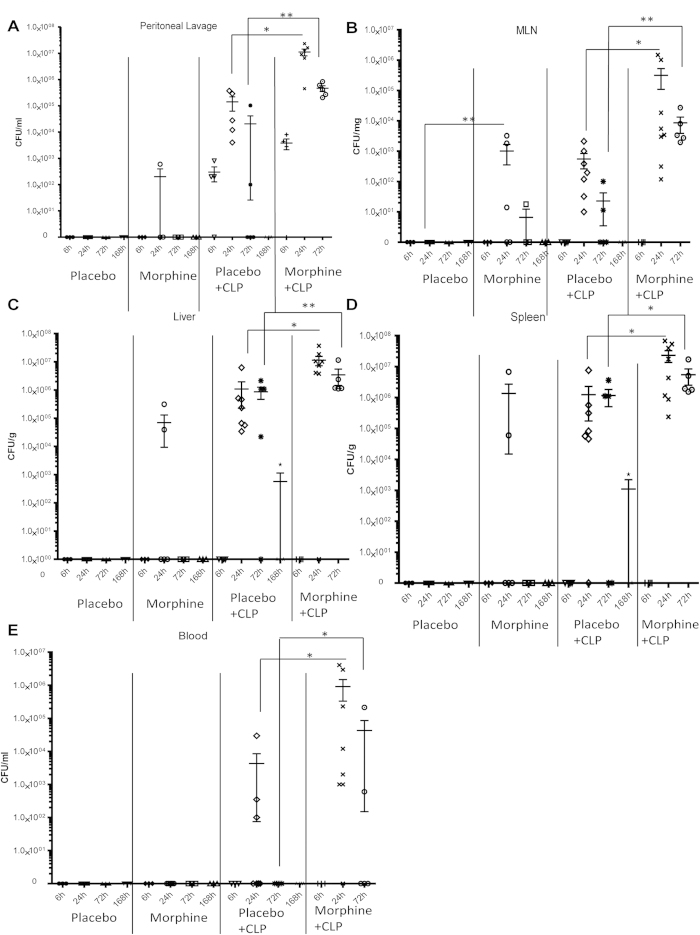
Morphine inhibits bacterial clearance and promotes bacterial dissemination during sepsis. Wild type mice were treated with 25 mg morphine pellets following CLP procedure. (**a**) Peritoneal lavage was collected at different time points and cultured on blood agar plates overnight. Bacterial colonies were quantified and described as colony-forming unit (CFU). (**b**) Bacterial colonies of MLN homogenates (**c**) Bacterial colonies of liver homogenates. (**d**) Bacterial colonies of spleen homogenates. (**e**) Bacterial colonies of whole blood. *P < 0.05 compared with placebo-treated animals ** p < 0.01 compared with placebo-treated animals (Mann-Whitney U test) (Each dot represents one animal).

Next, the bacterial load was determined in the peritoneal lavage fluid, mesenteric lymph node (MLN), liver, spleen, and blood at different time points following CLP in the presence or absence of morphine. As shown in Fig. 2, at 24 hours, morphine alone induced bacterial translocation into the peritoneal cavity, MLN, liver, and spleen, confirming that morphine itself can compromise the gut epithelial barrier, as previously reported^8^. In the placebo-treated CLP animals, the bacterial load in the peritoneal lavage fluid, MLN, liver, spleen, and blood reached its highest levels at 24 hours, and subsequently decreased at 72 hours. At 168 hours after the CLP, almost all bacteria that disseminated into the peritoneal organs were cleared in the placebo-treated animals. In the morphine-treated CLP mice, a significant increase in the amount of bacteria was observed in the peritoneal organs and blood compared with the placebo-treated mice at both 24 and 72 hours, indicating that morphine promoted bacterial dissemination and inhibited bacterial clearance. Bacterial translocation in liver homogenates was further evaluated using bacterial 16 s ribosomal DNA (rDNA). Consistent with data obtained using bacterial cultures and colony forming units, the bacterial load as measured by 16 s rDNA was also significantly higher in the morphine treated-CLP animals than all other groups (Supplementary Fig. 1). Because none of the animals survived beyond 96 hours in the morphine-treated CLP group, data regarding the bacterial load were not available for the last time point.

### Morphine treatment promotes Gram-positive bacterial dissemination and modulates the gut microbiome

**Figure 3:**
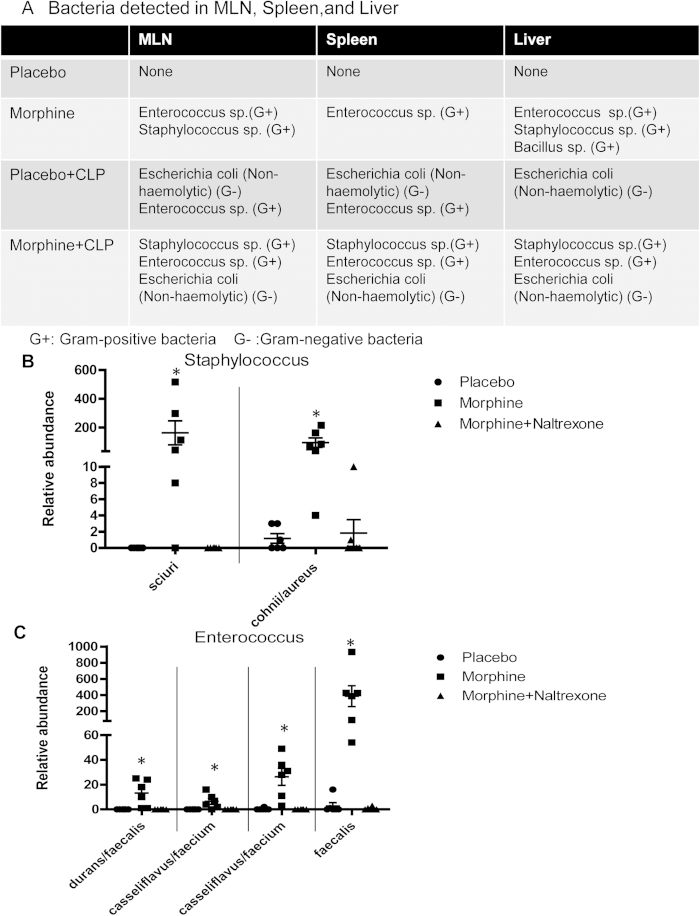
Morphine promotes Gram-positive bacterial translocation and modulates gut microbiome. (**a**) Bacterial families in MLN, spleen, and liver isolates from sham-operated or CLP mice treated with placebo or 25 mg morphine pellet. (**b**)–(**c**) Bacterial species identified in fecal contents from placebo or 25 mg morphine pellet-treated mice. *p < 0.05 compared with placebo-treated animals and morphine + naltrexone-treated animals (Each dot represents one animal).

We next serotyped the bacterial species that disseminated into the MLN, liver, and spleen following CLP. In placebo-treated CLP animals, the most common bacterial species detected in the MLN, spleen, and liver were non-hemolytic Escherichia coli (Fig. 3a), which are common Gram-negative commensal bacteria resident in the gut lumen; very few Enterococcus species were detected in the MLN and spleen. However, all MLN, spleen, and liver isolates from morphine-treated animals with or without CLP procedures revealed a significant prevalence of the Gram-positive families *Staphylococcus* and *Enterococcus* (Fig. 3a). These data were validated using Illumina sequencing of 16 S rDNA from liver samples of morphine-treated CLP mice, which showed a prevalence of both the *Enterobacteriaceae* and *Enterococcus* families (Supplementary Fig. 2a).

Analysis of the gut microbiome showed that morphine treatment induced enrichment of mostly the Firmicutes phylum and specifically the Gram-positive bacterial species *Staphylococcus sciuri*, *Staphylococcus cohnii*, and *Staphylococcus aureus* as well as *Enterococcus durans*, *Enterococcus casseliflavus*, *Enterococcus faecium*, and *Enterococcus faecalis* in the gut microbiome (Fig. 3b,c). Interestingly, these species belonged to the same families that were observed to translocate to the peritoneal organs following morphine treatment. Morphine-induced alterations of the gut microbiome were antagonized by the opioid receptor antagonist naltrexone (Fig. 3b,c), further validating that morphine treatment modulated the gut microbiome and thereby influenced the outcome of poly-microbial sepsis in an opioid receptor-dependent manner. This was also reflected in the bacterial sequences obtained from liver samples. When the beta-diversity of 16 S rDNA sequences from liver samples were compared between Placebo-treated (P), Placebo-treated CLP (PC), Morphine-treated CLP (MC), and Morphine + Naltrexone with CLP (MN) animals, the analysis showed that the MC animals clustered distinctly from the P and PC animals. However, morphine animals that were treated with naltrexone clustered with the PC group (Supplementary Fig. 2b). In summary, the data demonstrate a shift in the bacterial community in both the gut and within the translocated population in the liver following morphine treatment, which prompted us to investigate the effects on the subsequent immune responses.

### Morphine up-regulates IL-17A production during sepsis

**Figure 4:**
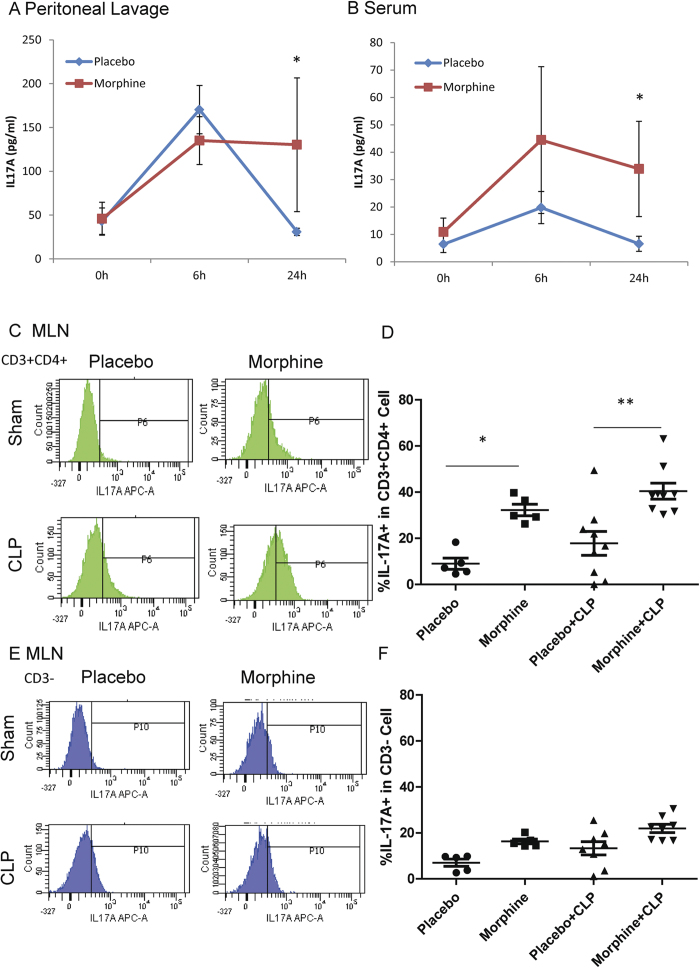
Morphine up-regulates IL-17A production during sepsis. (**a**) IL-17A concentrations in peritoneal lavage at different time points following CLP. *p < 0.05 (Student’s t test) (n = 5) (**b**) IL-17A concentrations in serum at different time points following CLP. *p < 0.05 (Student’s t test) (n = 5) (**c**) IL-17A expression in MLN CD3 + CD4 + cells. (**d**) Frequencies of IL-17A positive cells in CD3 + CD4 + cells. *p < 0.05, **p < 0.01 (ANOVA followed by Bonferroni’s t test) (Each dot represents one animal) (**e**) IL-17A expression in MLN CD3-cells (**f**) Frequencies of IL-17A positive cells in CD3- cells. (Each dot represents one animal).

The serum, peritoneal lavage fluid and MLN were collected to measure IL-17A production following CLP. The results show that IL-17A levels in the peritoneal lavage and serum increased 6 hours post-CLP in both placebo and morphine-treated mice. In placebo-treated mice, IL-17A concentrations were reduced to baseline levels at 24 hours, whereas the morphine-treated group sustained a high level of IL-17A (Fig. 4a,b). Previous studies have demonstrated two sources of IL-17A in response to infections: the T helper 17 cells and innate lymphoid cells^14^. To determine the type of cells that were producing IL-17A following the CLP procedure, we used anti-CD3 and anti-CD4 antibodies to separate MLN cells into two populations: CD3 + CD4 + T helper cells and CD3- non-T Cells (Supplementary Fig. 3). Flow cytometric analysis showed that placebo-treated animals did not exhibit any significant up-regulation of IL-17A at 24 hours after CLP (Fig. 4c,e). By contrast, a significant increase in IL-17A production was observed in the morphine-treated animals. Further characterization revealed that the major source of IL-17A in the MLN was the CD3 + CD4 + T helper cells (Fig. 4c,d) but not CD3- non-T cells (Fig. 4e,f).

### Neutralization of IL-17A improves the survival rate and attenuates sustained inflammation in CLP mice treated with morphine

**Figure 5:**
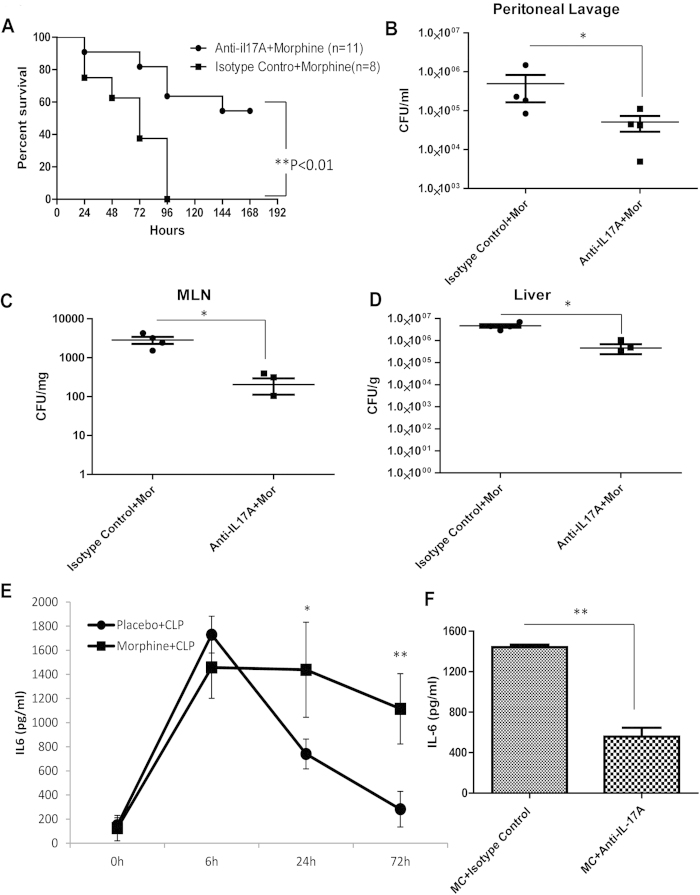
Neutralization of IL-17A improves survival rate and attenuates sustained inflammation in CLP mice treated with morphine **(a**) Kaplan–Meier plots of morphine-treated CLP mice injected with isotype control IgG or anti-IL17A antibody. p < 0.01 compared with anti-IL17A-treated mice subjected to CLP (Mantel-Cox log rank test) (**b**) Bacterial colonies of peritoneal lavage (**c**) Bacterial colonies of MLN (**d**) Bacterial colonies of liver *p < 0.05 (Mann-Whitney U test) (Each dot represents one animal) (**e**) IL-6 concentrations in serum at different time points following CLP. *p < 0.05, **p < 0.01 (Student’s t test) (n = 4) (**f**) IL-6 concentrations in serum in morphine-treated CLP mice injected with isotype control IgG or anti-IL-17A antibody. **p < 0.01 (Student’s t test) (n = 4).

To investigate the role of IL-17A in sepsis progression, a neutralizing antibody to IL-17A or its isotype control IgG was administered to morphine-treated CLP mice. As shown in Fig. 5a, the survival rate at 96 hours after CLP was significantly improved by anti-IL-17A, whereas none of the isotype control IgG injected mice survived. On day 7, the survival rate of mice treated with anti-IL-17A was 54.5% (Fig. 5a). Additionally, mice treated with anti-IL-17A showed significantly reduced bacterial disseminations to peritoneal organs at 24 hours following CLP compared with isotype-control treated animals (Fig. 5b–d), implying that neutralization of IL-17A following the CLP procedure improved gut barrier functions. It has been reported that the serum level of IL-6 is a good marker for severity during sepsis^15^. Thus, we determined the IL-6 levels in the serum at different time points following CLP. ELISA results showed that in placebo-treated animals, serum IL-6 peaked at 1729.6 pg/ml at 24 hours after CLP and then was reduced to baseline levels at 72 hours, whereas morphine-treated mice showed sustained high levels of IL-6 in the serum, even at 72 hours (Fig. 5e). Moreover, neutralization of IL-17A significantly decreased the IL-6 serum levels in the morphine-treated animals at 72 hours after CLP (Fig. 5f), validating the pro-inflammatory role of IL-17A during sepsis progression.

### High levels of IL-17A compromise gut epithelial barrier function and increase gut permeability

**Figure 6:**
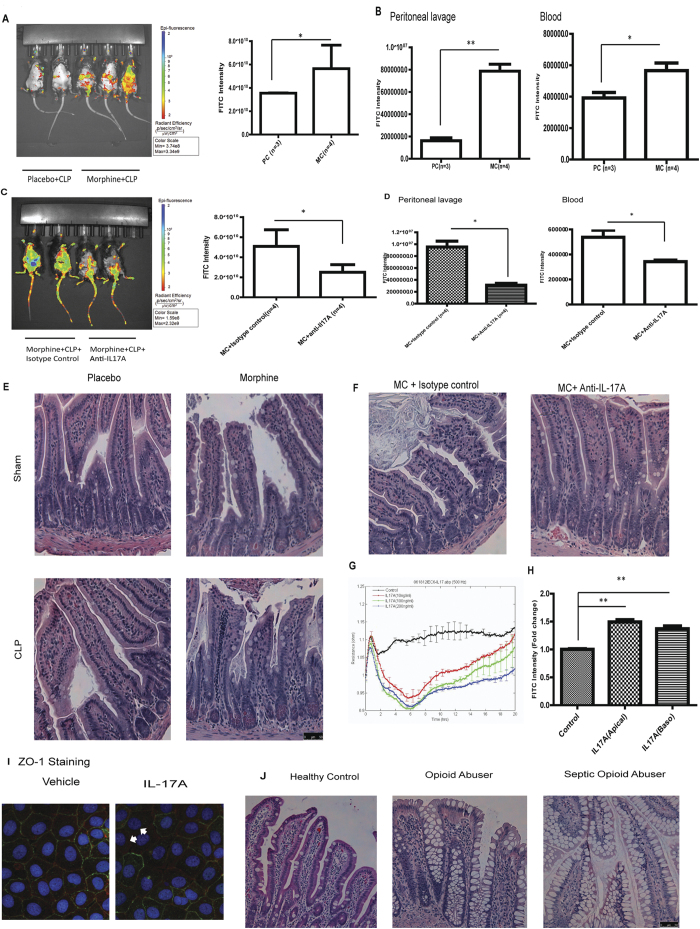
High levels of IL-17A compromises gut epithelial barrier function and increases gut permeability. (**a**) Morphine treatment increased FITC-dextran diffusion across the gut epithelium. The right panel was quantification of FITC intensity. *p < 0.05 (Student t test) (**b**) Quantification of FITC intensity in peritoneal lavage and whole blood *p < 0.05 (Student’s t test) (**c**) Anti-IL-17A injection reduced FITC-dextran diffusion across the gut epithelium in morphine-treated CLP animals. The right panel was quantification of FITC intensity. *p < 0.05 (Student’s t test) (**d**) Quantification of FITC intensity in peritoneal lavage and whole blood *p < 0.05 (Student’s t test) (**e**) H&E sections of small intestines from sham-operated or CLP animals treated with morphine or placebo. (**f**) H&E sections of small intestines from morphine-treated CLP animals injected with isotype control and anti-IL-17A. (**g**) TER was decreased by IL-17A in IEC-6 cell monolayer (**H**) The permeability of IEC-6 cell monolayer was increased in transwell system **p < 0.01 (ANOVA followed by Bonferroni’s t test) (n = 3) (i) ZO-1 organization in IEC6-cell monolayer treated by vehicle or 100 ng/ml IL-17A. Blue:DAPI Red:F-actin Green:ZO-1 White arrow indicates ZO-1 disruption.(**j**) H&E sections of small intestines from human patients.

Our results show that morphine induced significant IL-17A up-regulation in MLN CD4 + T cells, and because 80 ± 17.4% of the IL-17 + cells in the MLN express α4β7 integrin (Supplementary Fig. 4), a gut homing molecule that mediates lymphocyte recruitment to the intestines, we hypothesize that the IL-17A + T cells in the MLN migrate to intestinal tissues and modulate gut epithelial barrier function in morphine-treated CLP animals. To determine the gut permeability during sepsis progression, we gavaged mice with FITC labeled dextran and tracked the diffusion of the dextran. As shown in Fig. 6a, morphine treatment resulted in an increase in FITC-dextran diffusion across the gut epithelium, indicating that morphine increased gut permeability during sepsis. In morphine-treated CLP animals, FITC intensities were significantly higher in both the peritoneal lavage fluid and blood compared with placebo-treated animals, further validating the effects of morphine on gut permeability (Fig. 6b). In morphine-treated CLP animals that were injected with anti-IL-17A antibodies, we observed a decrease in FITC-dextran diffusion into the peritoneal cavity and blood (Fig. 6c,d), demonstrating that neutralization of IL-17A restored gut barrier function in morphine-treated CLP animals. To investigate the morphology of the intestinal epithelium in CLP animals, the small intestine and colon were excised, fixed and stained with hematoxylin and eosin (H&E). Histological analysis indicated severe epithelial injury in the small intestinal villi of morphine-treated CLP mice compared with the appearance of continuous epithelial cells lining gut mucosal surfaces in placebo-treated mice (Fig. 6e). Interestingly, the morphology of the colonic epithelium was not affected by morphine treatment (Supplementary Fig. 5), suggesting differential sensitivities of colonic and small intestinal epithelial cells to inflammatory stimulation. Neutralization of IL-17A also protected the epithelial structure of the small intestines in morphine-treated mice following CLP (Fig. 6f). To determine the direct effects of IL-17A on small intestinal epithelial cells, we determined the barrier function of IEC-6 cells by electrical cell impedance sensing (ECIS) arrays. The IEC-6 cell monolayers were treated with different concentrations of IL-17A (Fig. 6g). The TER values were significantly reduced following IL-17A treatment, and the duration of the effects of IL-17A persisted for a longer time with increasing concentrations. We further determined the barrier function of IEC-6 cells using a trans-well system. The tans-well assay showed that both apical and basolateral stimulation with IL-17A increased the permeability of the IEC-6 monolayer (Fig. 6h). We next investigated the organization of tight junction proteins, which play an important role in modulation of the epithelial barrier function between the epithelial cells. Staining of the tight junction protein zona occludens 1 (ZO-1) in IEC-6 cells also indicated the disruptive effects of IL-17A on ZO-1 organization (Fig. 6i). ZO-1 (green) co-localized with F-actin (red) on the apical side of the membrane in vehicle-treated cells, and its organization was disrupted following IL-17A stimulation. Consistent with the animal studies, we showed that the morphology of the small intestinal villi in human patients with opioid addiction was dramatically different than that of healthy controls. The structures of the small intestinal epithelium were even more severely disrupted in opioid-using septic patients, which again was consistent with our observations in mice (Fig. 6j).

### Gram-positive bacteria stimulates the MLN to produce IL-17A in a TLR2-dependent manner

**Figure 7:**
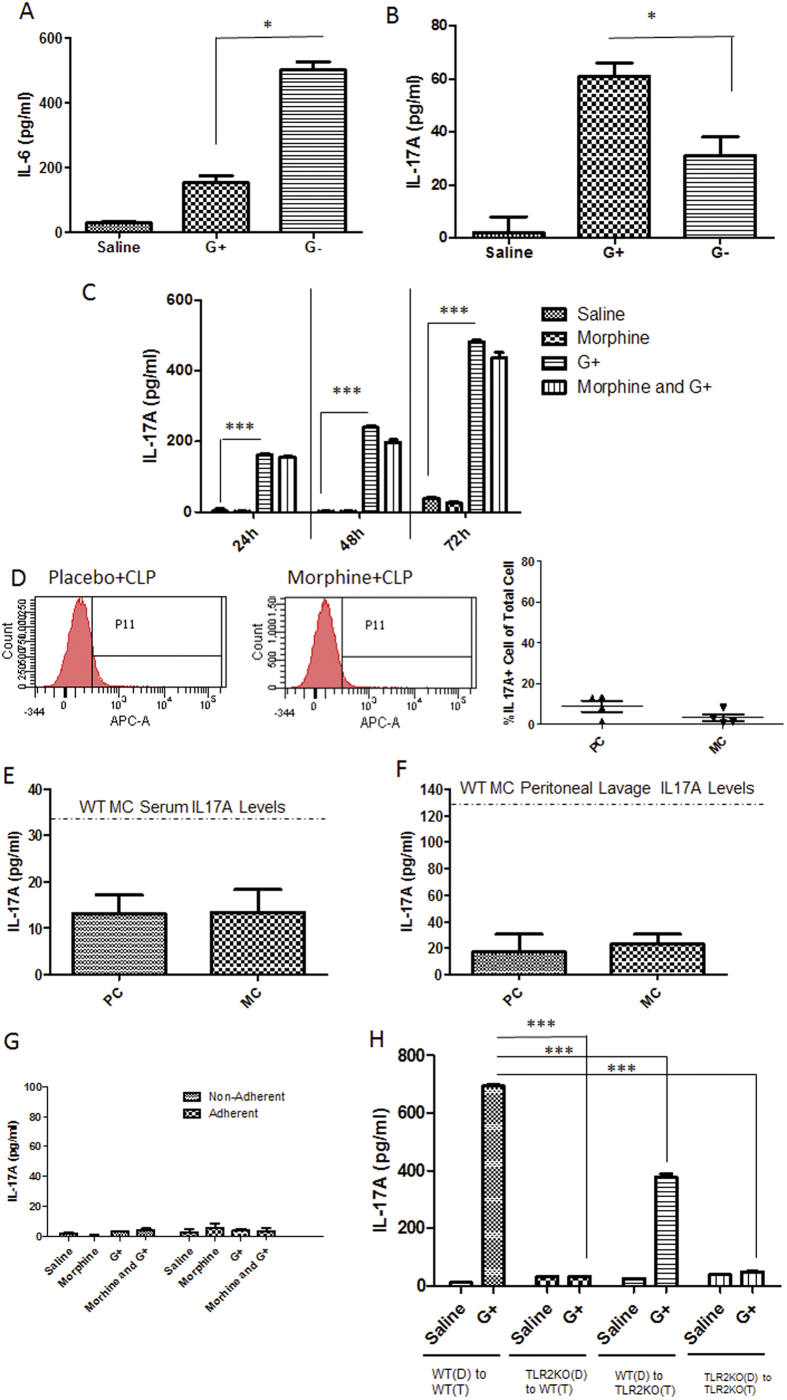
Gram-positive bacteria stimulate MLN to produce IL-17A in a TLR2-dependent manner. IL-6 (**a**) and IL-17A (**b**) concentrations of MLN cell supernatant stimulated by Gram-positive (G+) or Gram-negative (G-) bacteria. *p < 0.05 (ANOVA followed by Bonferroni’s t test) (n = 3) (**c**) IL-17A concentrations of MLN supernatant treated with morphine or Gram-positive (G+) bacterial mixture ***p < 0.001 (ANOVA followed by Bonferroni’s t test) (n = 3) (**d**) IL-17A expression in MLN cells from TLR2KO mice. The right panel is the frequencies of IL-17A positive cells in MLN from TLR2KO mice. PC: placebo + CLP; MC: morphine + CLP (**e**) IL-17A concentrations in serum in TLR2KO mice. PC: placebo + CLP; MC: morphine + CLP (n = 3) Dash line: Serum IL-17A concentration of wild type CLP mice treated with morphine (**f**) IL-17A concentrations in peritoneal lavage in TLR2KO mice. PC: placebo + CLP MC: morphine + CLP (n = 3) Dash line: Peritoneal lavage IL-17A concentration of wild type CLP mice treated with morphine (**g**) IL-17A concentrations in supernatant of adherent and non-adherent cells from MLN following Gram-positive (G + ) bacteria stimulation. (n = 3) (**h**) IL-17A concentrations in supernatant of non-adherent cells from MLN of WT or TLR2KO mice co-cultured with dendritic cells from blood of WT of TLR2KO mice. WT: wild type; TLR2KO: TLR2 Knock; D: dendritic cells; T: non-adherent T cells ***p < 0.001 (Two-way ANOVA followed by Bonferroni’s t test) (n = 3)

To investigate the mechanism by which morphine modulates IL-17A production in CLP animals, we cultured the immune cells from the MLN *in vitro* and stimulated the cells with either a Gram-negative bacteria species (*E coli*) or the Gram-positive species that translocated into the MLN following morphine treatment, which was a mixture of Enterococcus and Staphylococcus (Fig. 3a). The results showed that *E. coli* induced significant up-regulation of IL-6, whereas the mixture of *Enterococcus* and *Staphylococcus* induced significantly higher levels of IL-17A production (Fig. 7a,b). Interestingly, morphine treatment alone did not show any direct effects on IL-17A production by MLN cells (Fig. 7c). TLR2 has been shown to play an important role in recognizing cell wall components from Gram-positive bacteria and initiating the immune responses to pathogen stimulation. We measured IL-17A production in the serum, peritoneal lavage fluid, and MLN in TLR2 knockout (TLR2KO) mice following a CLP procedure. No IL-17A induction was observed in placebo or morphine-treated CLP TLR2KO animals, strongly implying that TLR2 plays a role in IL-17A responses in CLP-induced sepsis (Fig. 7d–f).

To understand the different roles of specific immune cells in the IL-17A response, we separated the adherent and non-adherent cells, as described previously^16^, and separately stimulated the cells with a Gram-positive bacterial lysate. No IL-17A induction was observed in the adherent or non-adherent cells (Fig. 7g), suggesting that antigen presenting cells are required for IL-17A responses. Because dendritic cells have been shown to initiate IL-17 responses^17^, we isolated non-adherent cells from the MLNs of WT or TLR2KO mice and co-cultured them with dendritic cells purified from the blood of WT or TLR2KO mice. Flow cytometric analysis indicated that approximately 80% of non-adherent cells in the MLN were CD3 + T cells (Supplementary Fig. 6). In response to bacterial stimulation, WT and TLR2KO T cells, which were co-cultured with WT dendritic cells, could produce high levels of IL-17A. Conversely, when TLR2KO T cells and WT T cells were co-cultured with TLR2KO dendritic cells, no significant IL-17A induction was observed (Fig. 7h), indicating that TLR2 expression on dendritic cells was an essential requirement for IL-17A production. Interestingly, the deficiency of TLR2s in T cells significantly attenuated IL-17A production (Fig. 7h), implying that TLR2s on T cells are also involved in the maximal IL-17A response.

### IL-1β and IL-23 promote IL-17A production by MLN Cells

**Figure 8:**
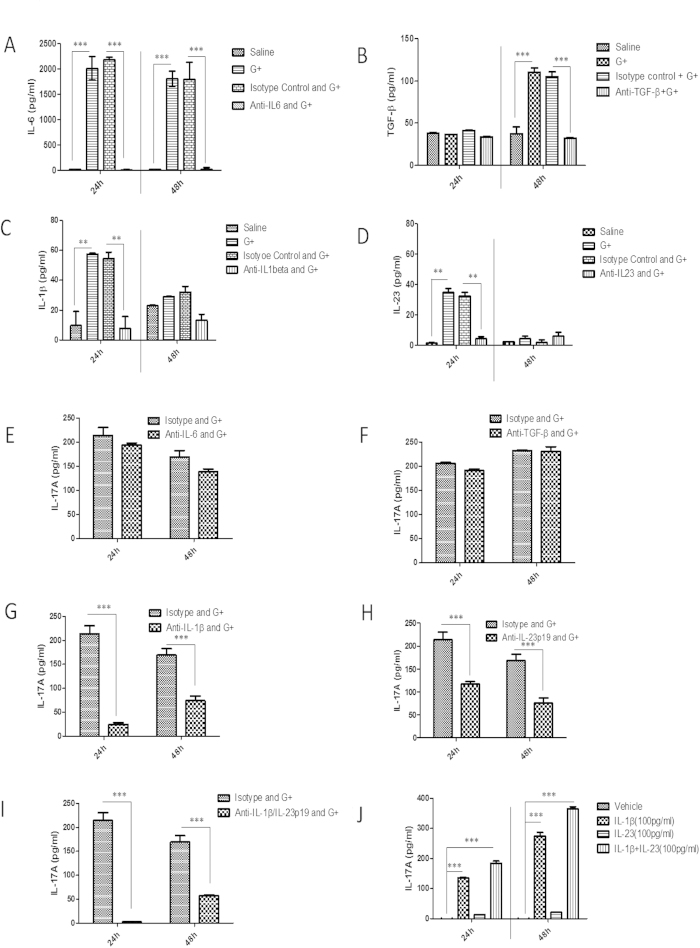
IL-1β and IL-23 promotes IL-17A production by MLN Cells (**a**)–(**d**) IL-6, TGF-β, IL-1β, and IL-23 concentrations of MLN adherent cell supernatant following Gram-positive (G+) bacterial stimulation ***p < 0.001 **p < 0.01 (ANOVA followed by Bonferroni’s t test) (n = 3) (**e**)–**(i**) IL-17A concentrations of MLN supernatant following G+ bacterial stimulation in the presence of isotype control, anti-IL-6, anti-TGF-β, anti-IL-1β or anti-IL-23p19 antibodies. ***p < 0.001 (ANOVA followed by Bonferroni’s t test) (n = 3) (**j**) IL-17A concentrations of MLN non-adherent cell supernatant following IL-1β or IL-23 stimulation. ***p < 0.001 (ANOVA followed by Bonferroni’s t test) (n = 3).

Previous studies have shown that IL-17A production is mediated by IL-6, TGF-β, IL-1β or IL-23 in different cell types^18^,^19^,^20^,^21^. To determine the role of each cytokine in IL-17A production in the MLN, we measured the concentrations of IL-6, TGF-β, IL-1β, and IL-23 in MLN adherent cell supernatant at different time points following bacterial stimulation. We observed significant up-regulation of IL-6, IL-1β, and IL-23 at 24 hours, which trended towards reduction after 48 hours. By contrast, no TGF-β up-regulation was observed until 48 hours after bacterial stimulation (Fig. 8a–d). We next determined the role of these cytokines in IL-17A production using cytokine neutralization antibodies (5 μg/ml) (Fig. 8e–i). As shown in Fig. 8e,f, neutralization of IL-6 or TGF-β did not affect IL-17A production. By contrast, neutralizing IL-1β and IL-23 inhibited IL-17A production with IL-1β showing a more dominant role in IL-17A modulation (Fig. 8g–i). Additionally, IL-1β alone was sufficient to induce IL-17A production, and the mixture of IL-1β with IL-23 showed synergistic effects on IL-17A induction in MLN cells (Fig. 8j), further validating the roles of IL-1β and IL-23 in IL-17A responses.

## Discussion

Many factors are independently associated with a greater risk of death attributed to sepsis, including co-morbid cancer, heart failure, and immunosuppression^1^. Several studies have indicated that morphine induces spontaneous sepsis in mice^8^ and that the opioid antagonist naltrexone blocks acute septic shock^22^. Although opioids are the mainstay for pain management in the ICU, their role in sepsis progression has not been investigated. In the present study, we demonstrated that two commonly prescribed opioids, morphine and methadone, significantly increased mortality rates in a murine model of poly-microbial sepsis, suggesting that the therapeutic window of opioids for pain management might be narrower in septic ICU patients. Clinical and laboratory studies provide strong evidence that opioids modulate immune function^5^,^8^,^23^,^24^,^25^,^26^, which is consistent with our current observation demonstrating decreased neutrophil recruitment into the peritoneal cavity in the early stages of sepsis (Supplementary Fig. 7) in morphine-treated animals. This observation implies that the defensive functions of immune cells for pathogen clearance are compromised in septic animals that are treated with morphine, resulting in a sustained higher bacterial load in different organs and the circulatory system. An analysis of gut microbial composition revealed an overgrowth of *Staphylococcus* and *Enterococcus* in the gut lumen following morphine treatment. Interactions between gut microbiota and the intestinal epithelial surface play important roles in the prevention of pathogenic bacterial outgrowth and maintaining gastrointestinal homeostasis^27^. Changes in composition or density of the microbiota may lead to higher susceptibility to a variety of pathogens and abnormal mucosal immune responses^28^. In our sepsis model, the overgrowth of *Staphylococcus* and *Enterococcus* in the gut lumen following morphine treatment resulted in Gram-positive bacterial dissemination. TLR2 activation by the disseminated Gram-positive bacteria induced the overexpression of the pro-inflammatory cytokine IL-17A. Enhanced IL-17A associated with morphine treatment in our sepsis model prompted us to investigate the role of IL-17A in sepsis progression.

IL-17A is a pro-inflammatory cytokine involved in the initiation and maintenance of several autoimmune disorders such as encephalomyelitis and inflammatory bowel disease^17^,^29^. One of the most important mechanistic attributes of IL-17A in this context is its modulation of the tight junction protein organization in endothelial cells, which thereby promotes blood-brain barrier disruption and central nervous system inflammation^30^,^31^. Increased IL-17 production has been shown to be associated with compromised gastro-intestinal integrity in pigtail macaques^32^. Our studies similarly show that overexpression of IL-17A during sepsis induces gut epithelial barrier dysfunction by disrupting the organization of tight junction proteins in gut epithelial cells. The role of IL-17A in these defects is supported by the observation that neutralization of IL-17A controlled the inflammation and improved survival following the CLP procedure, which was consistent with Flierl’s observation that IL-17A promoted inflammation and produced adverse functions in adult mice with severe sepsis^33^. However, contrasting studies show that IL-17 receptor signaling is required to control sepsis^34^,^35^. This inconsistency may be explained by the differences in the experimental approaches used: Deshmukh and Freitas used IL17R knockout mice or neutralized IL-17A in neonatal mice. The lack of IL-17 signaling in infant mice might result in compromised development of neutrophils and other immune cells, which may have contributed to the increased host susceptibility to infections.

The major source of IL-17A in our study was CD4 + helper T (TH) cells. To date, scientists have identified two different populations of TH_17_ cells: the inducible TH_17_ (iTH_17_) cells that arise from naive CD4 + T cells in response to antigen and cytokine stimulation and the natural TH_17_ (nTH_1_) cells that acquire the capacity to produce IL-17 during development in the thymus^18^. The nTH_17_ cells are poised to rapidly produce IL-17 upon stimulation without further differentiation in the peripheral tissues^36^ and show greater recruitment to Peyer’s patches and the lamina propria in the gut^37^. The presence of nTH_17_ cells may explain the quick IL-17A responses to bacterial stimulation in our study. Another novel observation in this study was that IL-17A production by the MLN following Gram-positive bacterial stimulation was independent of IL-6 and TGF-β, the key cytokines driving TH_17_ differentiation in previous publications^11^,^20^,^38^. In our sepsis model, dendritic cells expressing TLR2 were required for a robust IL-17A response, which was mediated by IL-1β and IL-23. Recent evidence suggests that the CD4 + T cells differentiate into more regulated classical TH_17_ cells in the presence of TGF-β. By contrast, the TGF-β independent pathway will generate an alternative cohort of TH_17_ cells, which are responsible for the excessive inflammation in many autoimmune disorders^17^. Our results corroborate this theory, and we show that IL-17A induction is not dependent on IL-6 or TGF-β; it instead requires IL-1β and IL-23 to produce pro-inflammatory IL-17A. The induction of pro-inflammatory IL-17A contributes to gut epithelial damage and compromised barrier function, leading to sustained bacterial dissemination and high levels of inflammation. γ/δT cells are also important sources of IL-17A, and these cells are particularly enriched at epithelial surfaces. Previous studies have shown that both lung γ/δ T cells and gut γ/δ intraepithelial lymphocytes can produce IL-17A, which plays an important role in the modulation of mucosal immunity^39^,^40^. In this study, the major cell type that produced IL-17A in the MLN was CD4 + T cells and only few γ/δT cells were detected in the MLN by flow cytometric analysis (Supplementary Fig. 8). In addition, we also observed that TLR2s on T cells were involved in the IL-17A response, which was consistent with Joseph Reynolds’s study: TLR2 agonists activated TLR2 signaling in CD4 + T cells and led to more robust proliferation and TH_17_ cytokine production, resulting in more severe pathology in autoimmune diseases such as EAE^19^. The present study lends further support to the pro-inflammatory role of IL-17A in the progression of sepsis. The advent of antibiotic resistance in various pathogens is a growing concern in clinics, and researchers have renewed their focus on developing novel anti-cytokine strategies for controlling infection-mediated hyper-inflammation rather than relying on antibiotics alone. However, no current large-scale trials of anti-cytokine molecules in the treatment of sepsis have achieved satisfying efficacy^41^. Therefore, our study may provide novel therapeutic targets to develop anti-cytokine strategies to control specific Gram-positive infections, especially in the context of opioid pain management.

To summarize, we report that opioid treatment in the context of sepsis results in the enrichment of the Gram-positive bacteria *Staphylococcus* and *Enterococcus* in the gut lumen and promotes dissemination. Disseminated Gram-positive bacteria induce IL-17A overexpression in a TLR2-dependent manner. Overexpression of IL-17A disrupts the gut barrier function and contributes to continued bacterial dissemination and sustained systemic inflammation, leading to a higher mortality rate. This study suggests that opioid administration will increase the risk of mortality associated with sepsis in ICU patients, and neutralization of IL-17A might be a novel strategy to control excess inflammation and improve survival in septic ICU patients who are receiving opioids for pain management (Supplementary Fig. 9).

## Methods

### Experimental animals and cell lines

Male C57BL/6 and TLR2KO mice were purchased from Jackson Laboratories (Bar Harbor, Maine, USA). The animal studies were approved by the Institutional Animal Care and Use Committee at the University of Minnesota (Protocol No. 1203A11091). All procedures were conducted in line with the guidelines set forth by the National Institutes of Health Guide for the Care and Use of Laboratory Animals.

Typically, 8–10 week old animals were used for our studies. IEC-6 cell lines were obtained from American Type Culture Collection and cultured as recommended by the supplier. IEC-6 cells are rodent small intestinal epithelial cell lines that have been used to study the intestinal barrier and integrity in previous publications^42^.

### Induction of sepsis using a CLP model and opioid treatment

Poly-microbial sepsis was induced as previously described^43^. Mice were anesthetized with 3% isoflurane, and a 1-cm midline incision was made on the anterior abdomen. The cecum was exposed and ligated, and the distance from the distal end of the cecum to the ligation point was approximately 1 cm. A double puncture was made with a 22-gauge needle to induce sepsis. The cecum was squeezed to allow the cecum contents to be expressed through the punctures. The cecum was then placed back in the abdominal cavity, and the peritoneal wall and skin incision were closed. All animals received 1 ml of saline by subcutaneous injection immediately after the surgery. Sham-operated animals (controls) underwent an identical laparotomy but without cecum ligation or puncture. The survival rates of animals were recorded every 12 hours until 7 days after the surgery.

For morphine treatment, a small incision was made at the dorsal torso of the mice. The placebo or a 25 mg slow release morphine pellet was inserted into the small pocket created during the incision, and the wound was closed using stainless steel wound-clips. Blood levels of morphine were approximately 718.42 ng/ml at 72 hours following pellet implantation (Supplementary Fig. 10), which were within the range observed in patients on opioids for moderate to severe pain (11 ng/ml to 1440 ng/ml)^44^. For methadone treatment, saline or 15 mg/kg of methadone was given by intraperitoneal injection. Methadone doses used in patients range from 50 to 150 mg/day^45^, which equates mouse doses of 10.28 mg/kg to 30.83 mg/kg according to the distinct Km factors of mice and humans^46^. Therefore, the dose of methadone used in our study was also within the physiological range observed in clinics.

For IL-17A neutralization, 100 μg of LEAF^TM^ anti-mouse IL-17A antibodies (BioLegend) were administered to mice by intraperitoneal injection every other day.

### Bacterial counts in the blood, peritoneal lavage fluid, MLN, liver and spleen

The bacterial counts were determined 6, 24, 72, and 168 hours after CLP surgery in the peritoneal lavage fluid, MLN, liver, spleen and blood. The peritoneal lavage fluid, blood, and homogenates of the liver, MLN, and spleen were cultured on a blood agar plate overnight. Bacterial colonies were quantified and described as colony forming units (CFU).

### Cytokine measurements

The cytokine levels were detected in the peritoneal lavage fluid, serum, and supernatants of MLN cells. The following mouse ELISA kits were used: IL-1β, IL-6, IL-23, IL-17A, and TGF- β (eBiosciences). All experiments were performed according to the manufacturer’s protocol.

### Flow cytometry analysis

The MLN was homogenized, and single cell suspensions were incubated with anti-CD3, anti-CD4, anti-IL-17A and anti-α4β7 antibodies following the manufacturer’s intracellular staining protocol (eBiosciences).

### Intestinal permeability

The animals were gavaged with 4 kD FITC-dextran (500 mg/kg body weight in a 50 mg/ml concentration) using a 4-cm long, curved needle with a plastic ball at the tip. The images of animals were analyzed with the Xenogen Spectrum system. After sacrifice, the blood and peritoneal lavage fluid were collected, and the intensity of FITC was determined with a fluorometer using an excitation wavelength of 488 nm and detecting the emission at 520 nm.

### Dendritic cell isolation

Dendritic cells from the blood of WT or TLR2KO mice were purified by a magnetic separation kit (Miltenyi Biotec) according to the manufacturer’s instructions.

### Measurement of trans-epithelial resistance

An ECIS 1600R (Applied BioPhysics, Troy, NY) was used to measure the trans-epithelial resistance (TER) of epithelial monolayers as described previously^47^. IEC-6 cells were seeded in the wells of the electrode array and grown to confluence. Then, the medium was changed, and the baseline TER was measured for 60 min to equilibrate the monolayers. Afterward, 400 ml of a medium containing different concentrations of IL-17A was applied to each well. The baseline TER of each experiment was normalized to 1.0 to enable comparisons and statistical analysis of TER changes over time following different treatments.

### Transwell assays

A transwell assay was performed as described previously^48^. IEC-6 cells were cultured in the top chamber of a transwell system, and 5 mg/ml 4 kD FITC-dextran was added to the top chamber after IL-17A (100 ng/ml) stimulation. The medium in the lower chamber was collected after 6 hours. The amounts of FITC-dextran in the lower chambers were determined with a fluorometer using an excitation wavelength of 488 nm and detecting emission at 520 nm.

### Immunofluorescence

IEC-6 cells in chamber slides were fixed with 1% paraformaldehyde in PBS for 10 min at room temperature. After washing in PBS and blocking nonspecific binding sites with 5% bovine serum albumin (BSA), the slides were incubated with polyclonal rabbit anti-ZO-1 (5 mg/ml, Invitrogen) in PBS with 5% bovine serum albumin (BSA) for 120 min at room temperature. After washing, the slides were incubated with rhodamine phalloidin (Invitrogen) and DyLightTM 488-conjugated AffiniPure Donkey anti-rabbit IgG (0.075 mg/ml, Jackson Lab, WestGrove, PA) for 60 min. The slides were then washed and mounted with coverslips using ProLong Gold anti-fade reagent with DAPI (Invitrogen). The slides were imaged with a confocal microscope (Nikon).

### Gut and liver microbiome analysis

The fecal content was collected from the gut region encompassing the distal cecum and approximately one inch of the colon. The gut tissue was washed and stored separately. The fecal matter was lysed using glass beads in MagnaLyser tissue disruptor (Roche), and the total DNA was isolated using a Power-soil/fecal DNA isolation kit (Mo-Bio) as per the manufacturer’s specifications. All samples were quantified via the Qubit® Quant-iT dsDNA Broad-Range Kit (Invitrogen, Life Technologies, Grand Island, NY), to ensure that they met the minimum concentration and mass for DNA, and submitted to Second genome Inc. for microbiome analysis as follows. To enrich the sample for the bacterial 16 S V4 rDNA region, DNA was amplified using fusion primers designed against the surrounding conserved regions, which are tailed with sequences to incorporate Illumina (San Diego, CA) flow cell adapters and indexing barcodes. Each sample was PCR amplified with two differently bar coded V4 fusion primers and was submitted for pooling and sequencing. For each sample, amplified products were concentrated using a solid-phase reversible immobilization method for the purification of PCR products and quantified by electrophoresis using an Agilent 2100 Bioanalyzer®. The pooled 16 S V4 enriched, amplified, barcoded samples were loaded into the MiSeq® reagent cartridge and then onto the instrument along with the flow cell. After cluster formation on the MiSeq instrument, the amplicons were sequenced for 250 cycles with custom primers designed for paired-end sequencing. Using QIIME and custom scripts, the sequences were quality filtered and demultiplexed using exact matches to the supplied DNA barcodes. The resulting sequences were then searched against the Greengenes reference database of 16 S sequences and clustered at 97% by uclust (closed-reference OTU picking). The longest sequence formed from each Operation Taxonomic Unit (OTU) was then used as the OTU representative sequence and assigned a taxonomic classification via the MOTHUR Bayesian classifier, which was trained against the Greengenes database clustered at 98%.

### Human samples and histology

Young adult (20–40 years old; de-identified) human tissue slides were commercially sourced from both the National Disease Resource Interchange as well as Bionet histology resources (University of Minnesota). Sections from patients with a history of intravenous use of heroin in addition to cocaine were compared with control sections. In total, 10 human samples were examined, four from healthy control, four from chronic opioid users and 2 from opioid abusers that have a diagnosis of sepsis. H&E staining was performed by the Comparative Pathology Shared Resource (CPSR) at the University of Minnesota, and slides were imaged using a Leica DM5500 B microscope. Representative images are shown. The Institutional Review Board (IRB) of the University of Minnesota determined that “This project does not meet the regulatory definition of human subject research. No further IRB review is required” under exemption 4.

### Statistical analysis

The data (except for the survival curves and scatter plots) are reported as the means ± SEMs of values of triplicate results. The means of different treatments were compared by Student’s t test or ANOVA followed by Bonferroni’s t test (GraphPad Prism Software). Bacterial counts were reported as the means of CFUs and were analyzed by the Mann-Whitney U test (GraphPad Prism Software). The survival rate was expressed as the percentage of live animals, and the Mantel-Cox log rank test was used to determine the differences between survival curves (GraphPad Prism Software version 3). A p value of 0.05 or less was considered significant. For the gut microbiome analysis, the Adonis test was used to determine significant whole microbiome differences among discrete categorical or continuous variables. In this randomization/Monte Carlo permutation test, the samples were randomly reassigned to the various sample categories, and the mean normalized cross-category differences from each permutation were compared to the true cross-category differences. The fraction of permutations with greater distinction among categories (larger cross-category differences) than those observed with the non-permuted data were reported as the p-value for the Adonis test.

## Additional Information

**How to cite this article**: Meng, J. *et al.* Opioid Exacerbation of Gram-positive sepsis, induced by Gut Microbial Modulation, is Rescued by IL-17A Neutralization. *Sci. Rep.* **5**, 10918; doi: 10.1038/srep10918 (2015).

## Supplementary Material

Supplementary Information
