# Supplementary material for: Opioid Exacerbation of Gram-positive sepsis, induced by Gut Microbial Modulation, is Rescued by IL-17A Neutralization
Source: Sci Rep. 2015 Jun 3;5:10918. doi: 10.1038/srep10918 (PMC4454150; doi:10.1038/srep10918)
Supplement: Supplementary Information [file srep10918-s1.pdf]

a) Title: Opioid Exacerbation of Gram-positive sepsis, induced by Gut Microbial Modulation, is Rescued by IL-17A Neutralization

b) Authors: Jingjing Meng[1], Santanu Banerjee[2], Dan Li[3], Greg Sindberg[4], Fuyuan Wang[4], Jing Ma[2], Sabita Roy* [1, 2]

[1]Department of Pharmacology, University of Minnesota Medical School, Minneapolis, Minnesota
[2]Department of Surgery, Division of Infection, Inflammation, and Vascular Biology, University of Minnesota Medical School, Minneapolis, Minnesota
[3]Department of Infectious Diseases, First Affiliated Hospital of China Medical University, Shenyang, China
[4]Department of Veterinary Population Medicine, College of Veterinary Medicine, University of Minnesota, St. Paul, Minnesota

c)Correspondence: Sabita Roy
Email: royxx002@umn.edu Phone: 612-624-4615 Fax Number: 612 626-4900

Supplementary Fig. 1

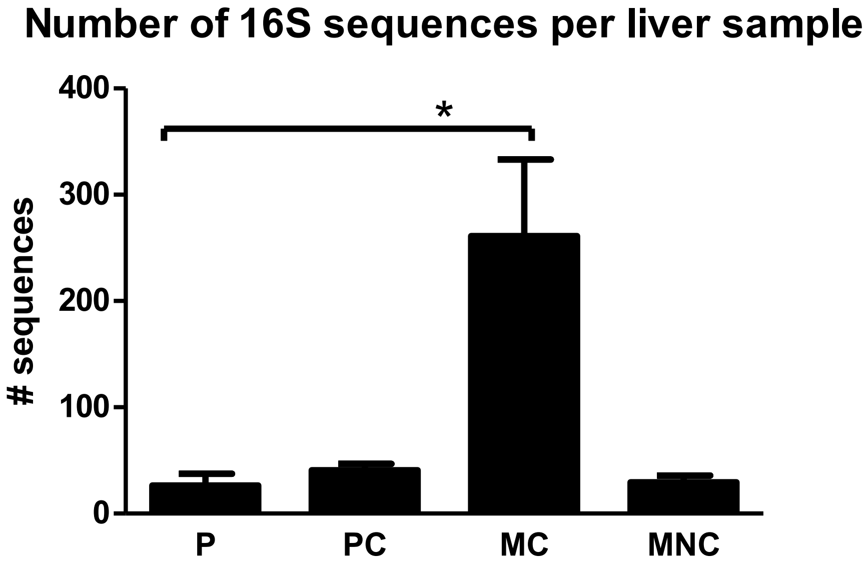

Supplementary Fig.1 DNA from Liver samples was collected and amplified for 16S V4 region and sequenced on Illumina platform. Total sequences are significantly higher in MC group, supporting bacterial translocation into the liver, which was antagonized by naltrexone treatment. P: Placebo, PC: Placebo+CLP, MC:Morphine+CLP, MNC:Morphine+Naltrexone+CLP, MC:N=2; all other groups, N=3. *P<0.05

Supplementary Fig. 2

A

Top Families in MC liver (>2% total)	Percent of Total MC Liver sequences
Enterobacteriaceae	76.43%
Lactobacillaceae	9.55%
Flavobacteraceae	5.10%
Streptococcaceae	5.10%
Ruminococcaceae	3.18%
Enterococcaceae	2.55%

B

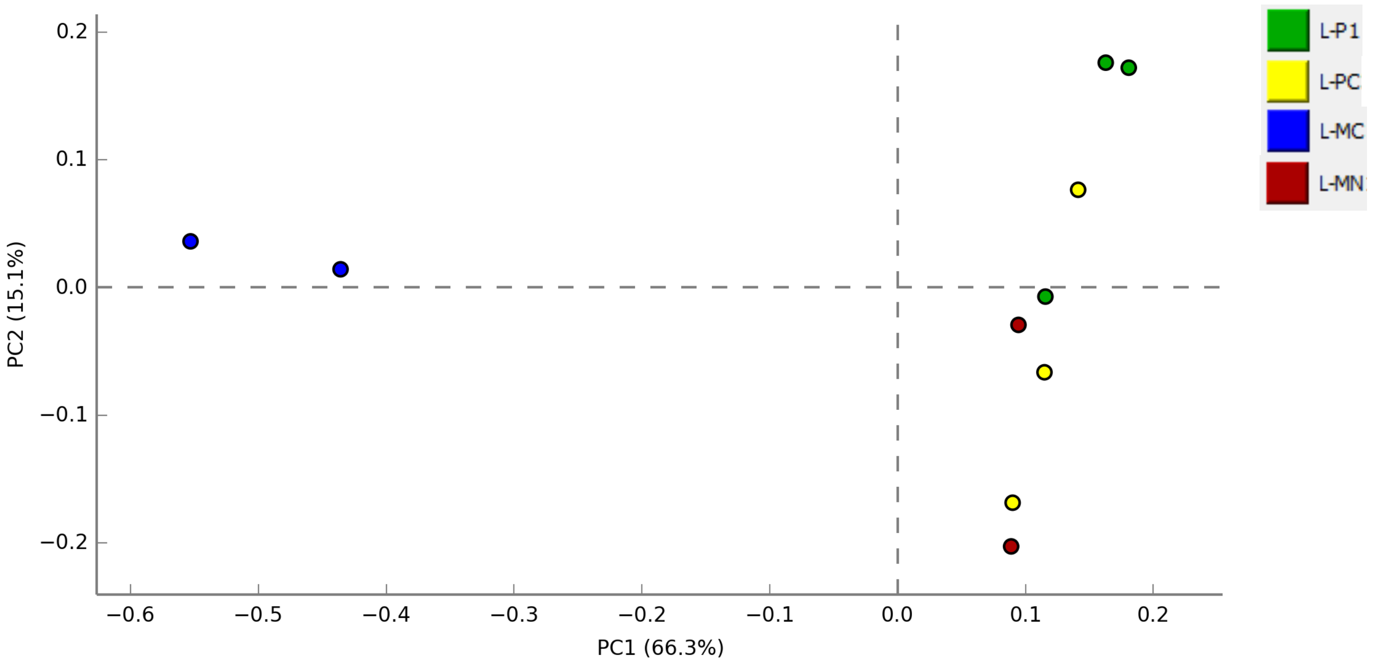

Supplementary Fig.2 (a)DNA from Liver samples was collected and amplified for 16S V4 region and sequenced on Illumina platform. OTUs were compared to GreenGenes 13.5 reference population for identification. MC: Morphine+CLP (b) Beta Diversity of 16S rDNA sequences from liver samples. MC samples cluster distinctly from other groups, which highlights that the composition of organisms found in morphine-treated CLP group differs from the placebo and naltrexone-treated groups. P: Placebo,PC: Placebo+CLP, MC:Morphine+CLP, MN:Morphine+Naltrexone+CLP

Supplementary Fig. 3

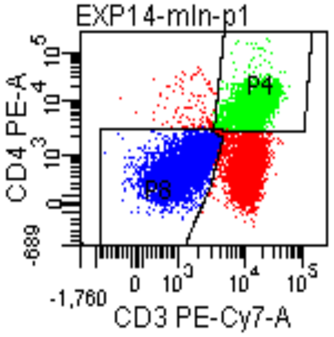

Supplementary Fig. 3 MLN cells were fixed and incubated with anti-CD3 and anti-CD4 antibodies and separated as two populations: CD3+CD4+ Cells and CD3- Cells.

Supplementary Fig. 4

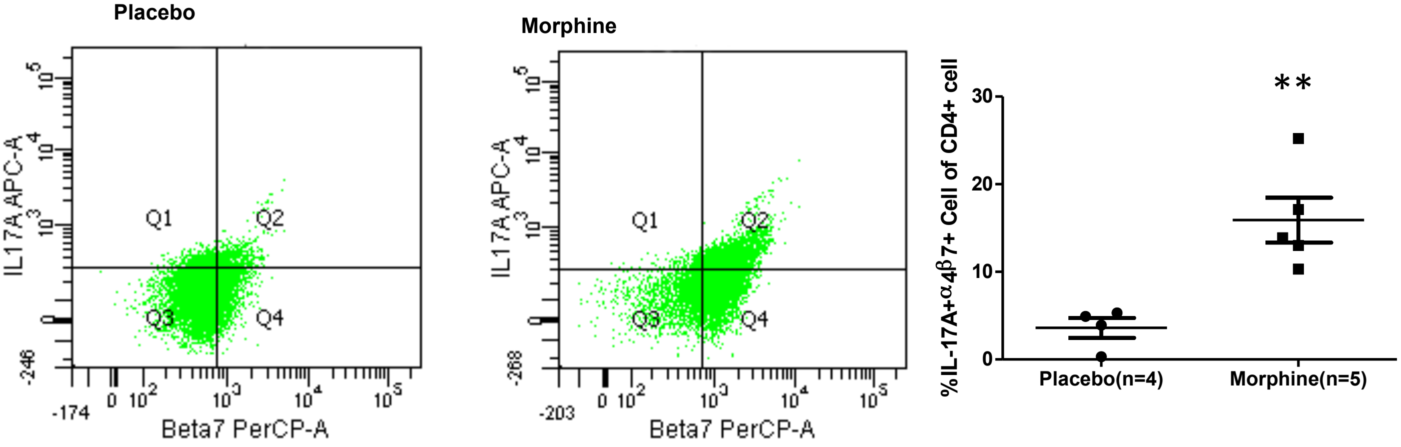

Supplementary Fig.4 CD3+CD4+ Cells in MLN were incubated with anti-IL17A and anti-α4β7 integrin antibodies. ** p<0.01 compared with placebo-treated animals (student t test).

Supplementary Fig.5

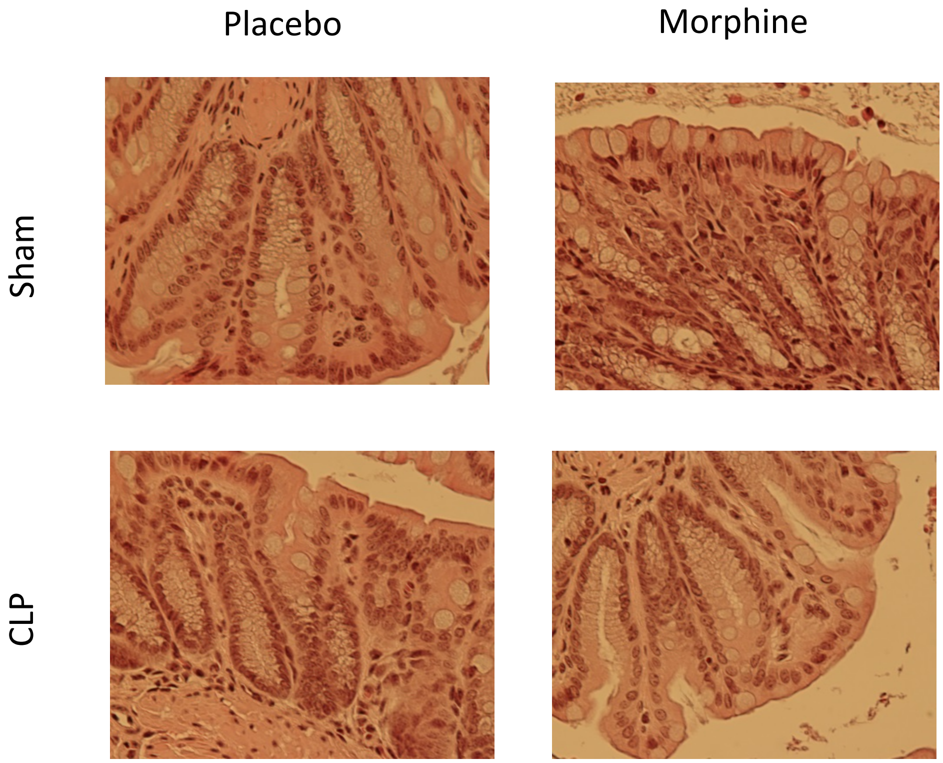

Supplementary Fig 5. H&E sections of colons from sham-operated or CLP animals treated with morphine or placebo.

Supplementary Fig.6

A Whole MLN cells Before separation

B MLN Non-adherent cells

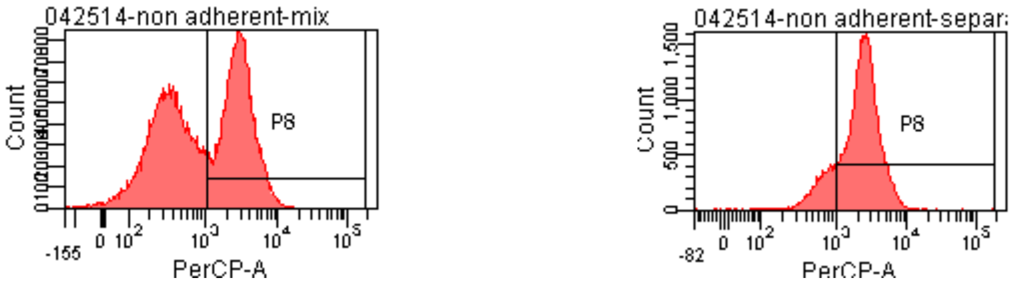

Supplementary Fig.6 MLN cells were fixed and incubated with anti-CD3 antibodies and separated as two populations: CD3+ Cells and CD3- Cells. After separation, approximately 80% of non-adherent cells were CD3+ T cells.

Supplementary Fig. 7

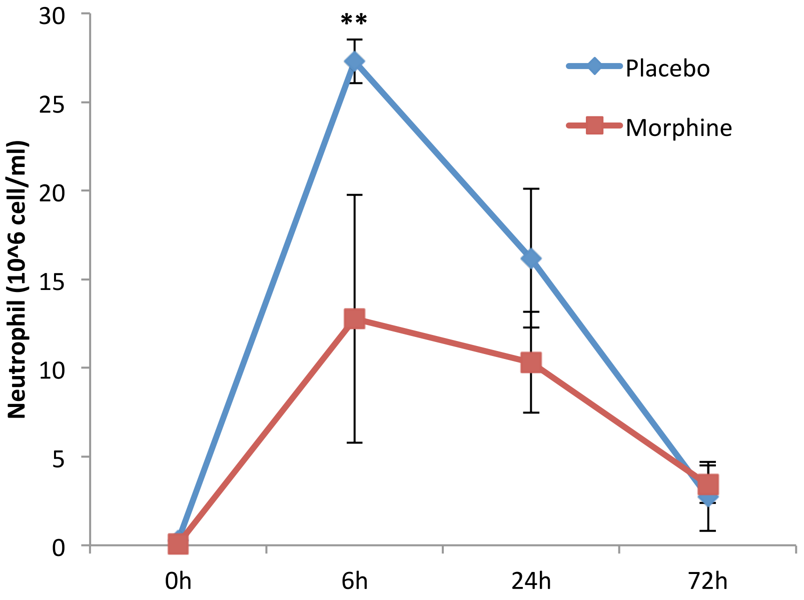

Supplementary Fig.7 The numbers of neutrophils in peritoneal lavage at different time points were determined by anti-neutrophil antibodies (Abcam) according to the manufacturer's instructions. **p<0.01 (Student t test)

Supplementary Fig. 8

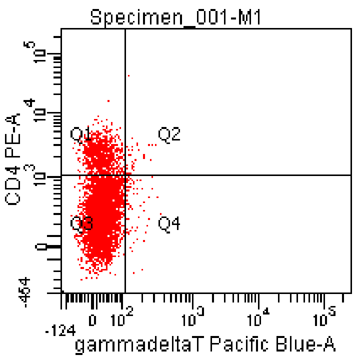

Supplementary Fig. 8 MLN cells were fixed and incubated with anti-γδ TCR and anti-CD4 antibodies.

Supplementary Fig.9

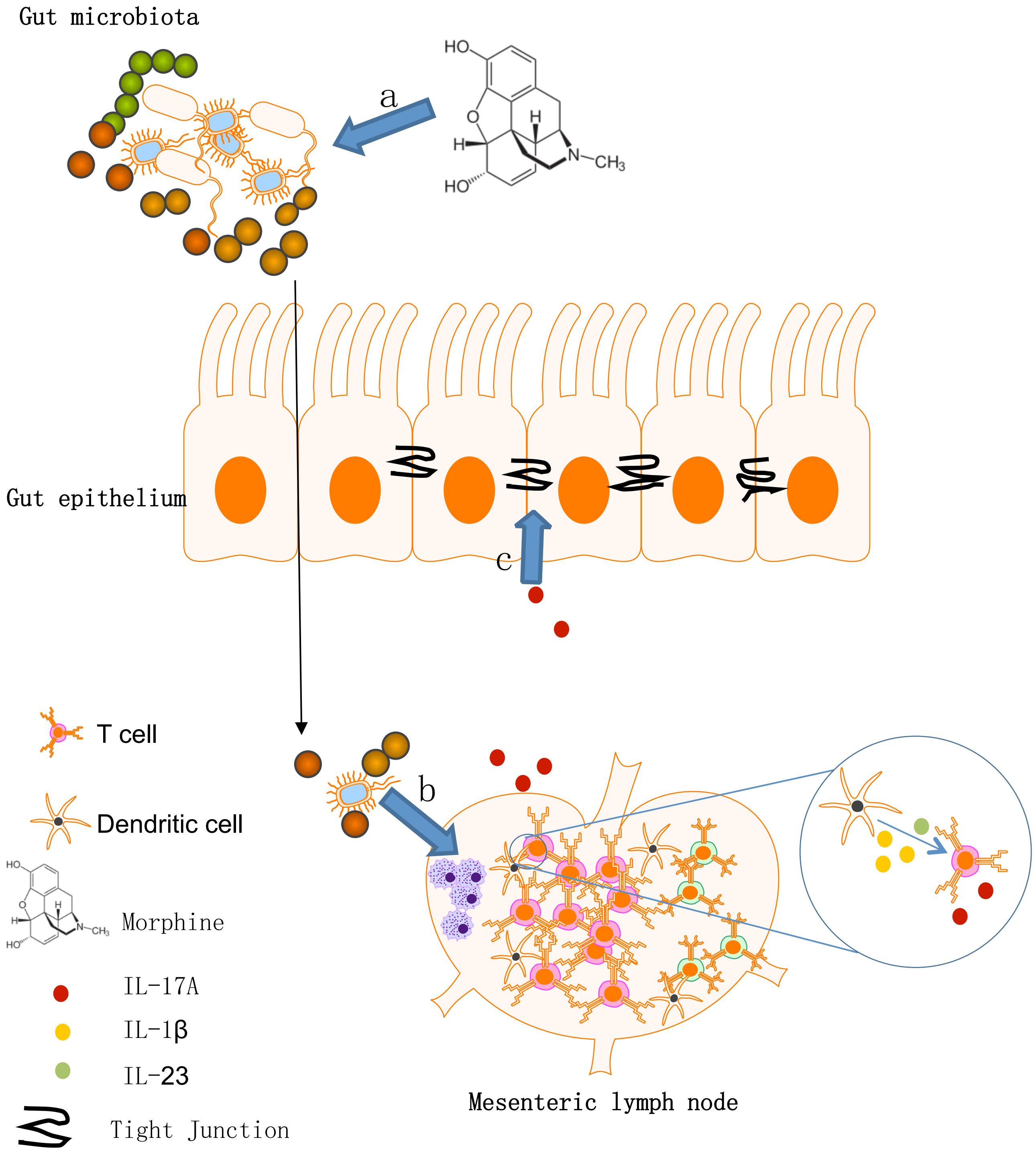

Supplementary Fig.9 Model of opioid modulation of sepsis progression. (a) Morphine treatment induces enrichment of Gram-positive bacteria *Staphylococcus* and *Enterococcus* in the gut lumen and promotes Gram-positive bacterial dissemination. (b) Disseminated Gram-positive bacteria induced IL-17A overexpression in a TLR2-dependent manner. (c) Excess IL-17A disrupted tight junction organization and increased gut permeability, which contributed to sustained inflammation and higher mortality rates associated with opioid treatment.

Supplementary Fig. 10

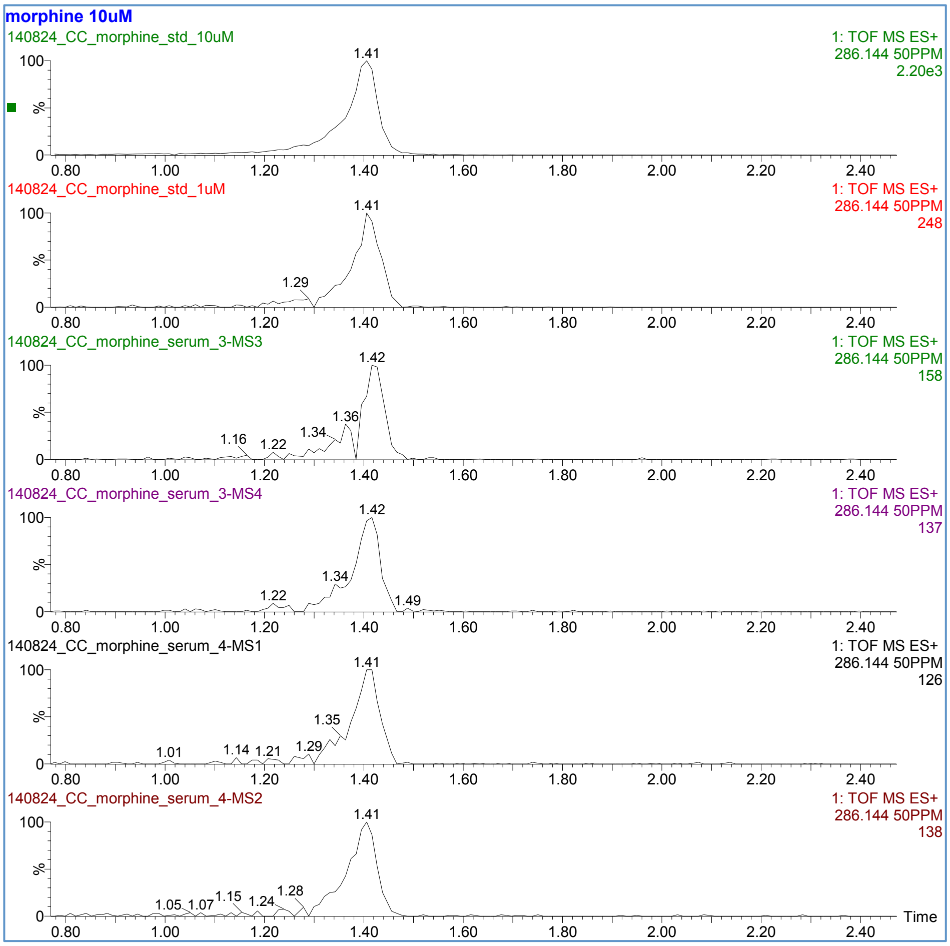

Animal ID	Morphine (ng/ml)
3-MS3	742.47327
3-MS4	697.339252
4-MS1	721.796489
4-MS2	712.075316
Average	718.421

Supplementary Fig.10 WT mice were treated with 25mg slow release morphine pellet subcutaneously and the serum samples were collected after 72 h of pellet implantation. Serum samples were precipitated by 4 volumes of 66% ACN. The supernatants were analyzed in a UPLC-SYNAPT Q-TOF system
